# Sophocarpine Suppresses NF-κB-Mediated Inflammation Both *In Vitro* and *In Vivo* and Inhibits Diabetic Cardiomyopathy

**DOI:** 10.3389/fphar.2019.01219

**Published:** 2019-10-31

**Authors:** Fang Zou, Ling Wang, Han Liu, Wei Wang, Longlong Hu, Xiaoying Xiong, Lijuan Wu, Yunfeng Shen, Renqiang Yang

**Affiliations:** ^1^Department of Endocrinology and Metabolism, The Second Affiliated Hospital of Nanchang University, Nangchang, China; ^2^Department of Clinical Laboratory, The Second Affiliated Hospital of Nanchang University, Nangchang, China; ^3^Department of Cardiovascular Disease, The Second Affiliated Hospital of Nanchang University, Nangchang, China

**Keywords:** sophocarpine, NF-κB, inflammation, diabetes, cardiomyopathy—diagnostics

## Abstract

Diabetic cardiomyopathy (DCM) is a leading cause of mortality in patients with diabetes. DCM is a leading cause of mortality in patients with diabetes. We used both *in vitro* and *in vivo* experiments to investigate the hypothesis that sophocarpine (SPC), a natural quinolizidine alkaloid derived from a Chinese herb, could protect against DCM. We used hyperglycemic myocardial cells and a streptozotocin (STZ)-induced type 1 diabetes mellitus mouse model. SPC protected myocardial cells from hyperglycemia-induced injury by improving mitochondrial function, suppressing inflammation, and inhibiting cardiac apoptosis. The SPC treatment significantly inhibited the activation of nuclear factor kappa-light-chain-enhancer of activated B cells (NF-κB) signaling in high-glucose-stimulated inflammatory responses. Moreover, SPC significantly slowed the development and progression of DCM in STZ-induced diabetic mice. These results show that SPC suppresses NF-κB-mediated inflammation both *in vitro* and *in vivo* and may be used to treat DCM.

## Introduction

Cardiovascular disease is a major cause of morbidity and mortality worldwide, especially for patients with diabetes (Tall and Levine, 2017; He et al., 2019; Tall and Jelic, 2019). Previous studies have reported that more than half of these patients die from diabetic cardiomyopathy (DCM), which is characterized by changes in myocardial structure and function such as ventricular hypertrophy, cardiac fibrosis, and heart failure (Ye et al., 2018; Kang et al., 2019; Knapp et al., 2019; Zheng et al., 2019). Due to the increased prevalence of DCM-related mortality, an understanding of the mechanism of DCM pathogenesis and effective therapies are needed urgently (Sun et al., 2019; Zheng et al., 2019; Wu et al., 2019b). The pathophysiology of DCM is complex; however, inflammatory upstream events that are mainly induced by hyperglycemia are important. The downstream events that eventually lead to heart failure include elevated oxidative stress, cardiac inflammation, cardiomyocyte apoptosis, interstitial fibrosis, and myocardial remodeling (Ye et al., 2018; Zhang et al., 2018a; Bombicz et al., 2019; Wu et al., 2019b). Therefore, suppressing inflammation is a promising strategy for treating DCM (Ye et al., 2018; Zheng et al., 2018; Bombicz et al., 2019; Wu et al., 2019b).

Sophocarpine (SPC) is a natural quinolizidine alkaloid derived from *Sophora flavescens*, a traditional Chinese herb that has been used to treat illnesses for nearly 2,000 years (Zhou et al., 2018; Wu et al., 2019a). SPC attenuates wear particle-induced implant loosening (Zhou et al., 2018), and both *in vitro* and *in vivo* studies suggest that it has strong anti-inflammatory and pharmacological effects on a variety of human diseases including viral infections, allergies, and cancer (Huang et al., 2016; Zhang et al., 2016; Sang et al., 2017; Zhu and Zhu, 2017; Liu et al., 2017b; Jiang et al., 2018; Li et al., 2018; Zhou et al., 2018; Zhang et al., 2018b; Wu et al., 2019a). Moreover, several previous studies reported that SPC can protect against cardiovascular diseases (Li et al., 2011; Zhang et al., 2012; Li et al., 2014). (Li et al., 2014) reported that oral SPC protected rat hearts against pressure-overload-induced cardiac fibrosis (Li et al., 2014). Zhang *et al.* reported that SPC attenuates the Na^+^-dependent Ca^2+^ overload induced by *Anemonia sulcata* toxin-increased late sodium current in rabbit ventricular myocytes (Zhang et al., 2012). In another study, administering SPC to rats preserved myocardial function following ischemia-reperfusion by inactivating nuclear factor kappa-light-chain-enhancer of activated B cells (NF-κB) (Li et al., 2011). However, it is unclear whether SPC has cardioprotective effects against DCM.

Because of its effect on inflammatory responses and cardioprotective properties, here we conducted both *in vitro* and *in vivo* experiments to explore: (1) the effect of SPC on high glucose (HG)-induced mitochondrial dysfunction, inflammation, apoptosis of myocardial cells; (2) the effect of SPC on collagen deposition, fibrosis, left ventricular remodeling and cardiac dysfunction in DCM mice; and (3) the underlying mechanism.

## Results

### SPC Protects Against HG-Induced Inflammatory Responses in Myocardial Cells

**Figure 1:**
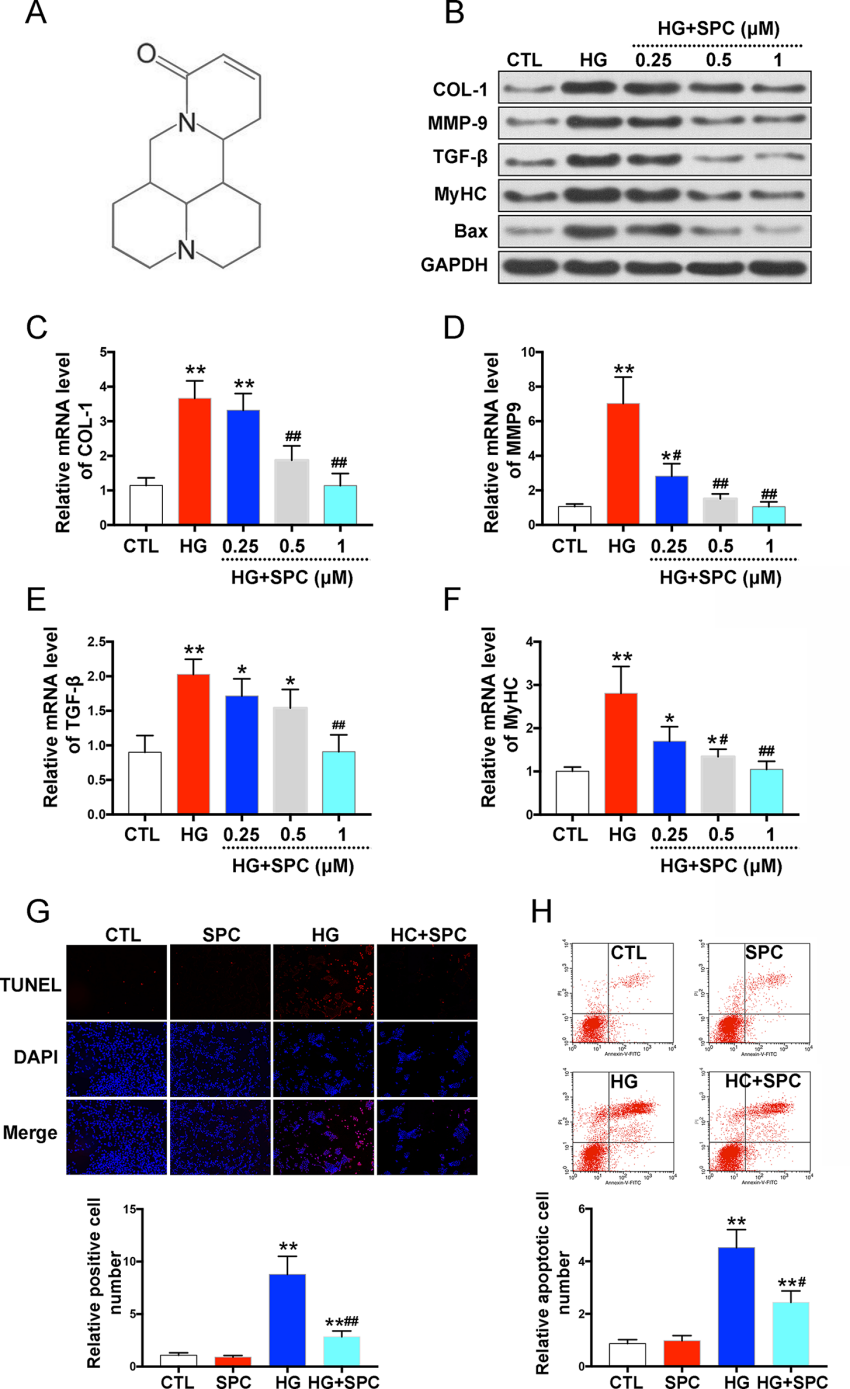
SPC protects against HG-induced inflammatory responses in H9c2 cells. **(A)** The chemical structure of SPC. **(B)** Western blot analysis showed that HG stimulation for 12 h remarkebly increased the expression of COL-1, MMP-9, TGF-β, MyHC, and Bax, which was then significantly inhibited by SPC in a dose dependent manner. **(C**–**F)** The results of qPCR further confirmed the findings of western blot analysis. **(G)** TUNEL staining showed that the increased apoptosis of H9c2 cells was effectively attenuated by SPC. Figures are magnified as 100×. **(H)** Flow cytometry assay confirmed the results of TUNEL staining. CTL, control group; SPC, Sophocarpine; HG, high glucose. *P < 0.05 when compared with the results of control group; **P < 0.01 when compared with the results of control group; ^#^P < 0.05 when compared with the results of HG group; ^##^P < 0.01 when compared with the results of HG group.

To investigate the cytotoxicity of SPC, H9c2 cells were treated with several doses (0–10 mM) (Zhou et al., 2018) of SPC for 48 and 96 h. As is shown in **Supplementary Figures 1**, no toxic effects of SPC were found on H9c2 cells, up to the maximal concentration of 10 mM. To assess the effect of SPC on HG-induced inflammatory responses, biomarkers of hypertrophy, cell fibrosis, and apoptosis were assessed by western blot assay after treatment. As in shown in **Figure 1**, HG stimulation for 12 h remarkably increased the expression of pro-fibrotic biomarkers including collagen 1 (COL-1), matrix metalloproteinase 9 (MMP-9), and transforming growth factor-β (TGF-β); hypertrophy biomarker myosin heavy chain (MyHC); and cell apoptotic biomarker Bax, which was then significantly inhibited by SPC in a dose dependent manner (**Figure 1B**). The results of qPCR further confirmed the findings of western blot analysis (**Figures 1C–F**). By conducting TUNEL (terminal deoxynucleotidyl transferase-mediated dUTP nick end labeling) staining, we found that the increased apoptosis of H9c2 cells was effectively attenuated by 1 mM SPC (**Figure 1G**), which was also confirmed by flow cytometry apoptosis assay (**Figure 1H**).

To confirm our findings about the effects of SPC on myocardial cells, we also applied additional experiments using neonatal mouse cardiomyocytes (NMCMs). As is shown in **Supplementary Figure 2**, SPC also attenuated HG-stimulated inflammatory responses and apoptosis in NMCMs, which was consistent with what we found in H9c2 cells.

### SPC Attenuated HG-Stimulated Mitochondrial Dysfunction in H9c2 Cells

**Figure 2:**
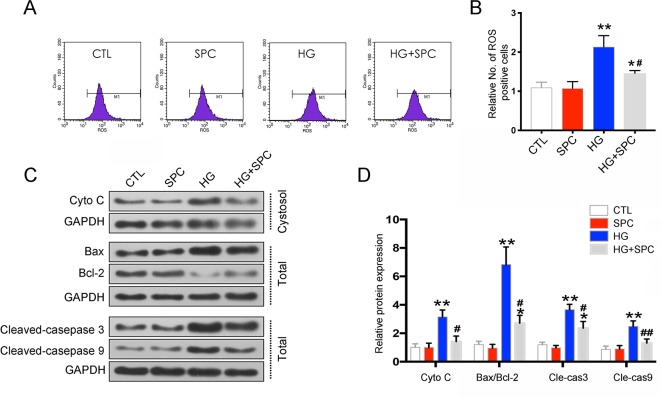
SPC attenuated HG-stimulated mitochondrial dysfunction in H9c2 cells. **(A** and **B)** Effects of SPC (1 mM) treatment on ROS production using flow cytometry assay. **(C)** Western blot analysis showed that HG induction for 12 h significantly increased cytochrome c release and caspase-3/9 activation, which was effectively inhibited by SPC. Moreover, HG stimulation upregulated the expression of pro-apoptotic Bax and downregulated the expression of anti-apoptotic Bcl-2, whereas these changes were attenuated by application with expression. **(D)** Quantification of the western blot analysis. CTL, control group; SPC, Sophocarpine; HG, high glucose, ROS, reactive oxygen species. *P < 0.05 when compared with the results of control group; **P < 0.01 when compared with the results of control group; ^#^P < 0.05 when compared with the results of HG group; ^##^P < 0.01 when compared with the results of HG group.

To uncover the possible underlying mechanism of the anti-apoptotic effect of SPC on H9c2 cells, the mitochondrial-mediated apoptotic pathway, which plays a vital role in HG-stimulated H9c2 cell apoptosis (Guo et al., 2018), was analyzed. As is shown in **Figure 2**, HG induction for 12 h significantly increased reactive oxygen species (ROS) production, which was effectively inhibited by 1 mM SPC treatment (**Figures 2A, B**). Similarly, the cytochrome c release and caspase-3/9 activation induced by HG were also inhibited by SPC. Moreover, we determined the effect of SPC on Bcl-2 family proteins expression. HG stimulation up-regulated the expression of pro-apoptotic Bax and down-regulated the expression of anti-apoptotic Bcl-2, whereas these changes were attenuated by application with expression (**Figures 2C, D**).

### SPC Suppressed the Activation of the NF-κB Signaling in HG-Stimulated Inflammatory Responses

**Figure 3:**
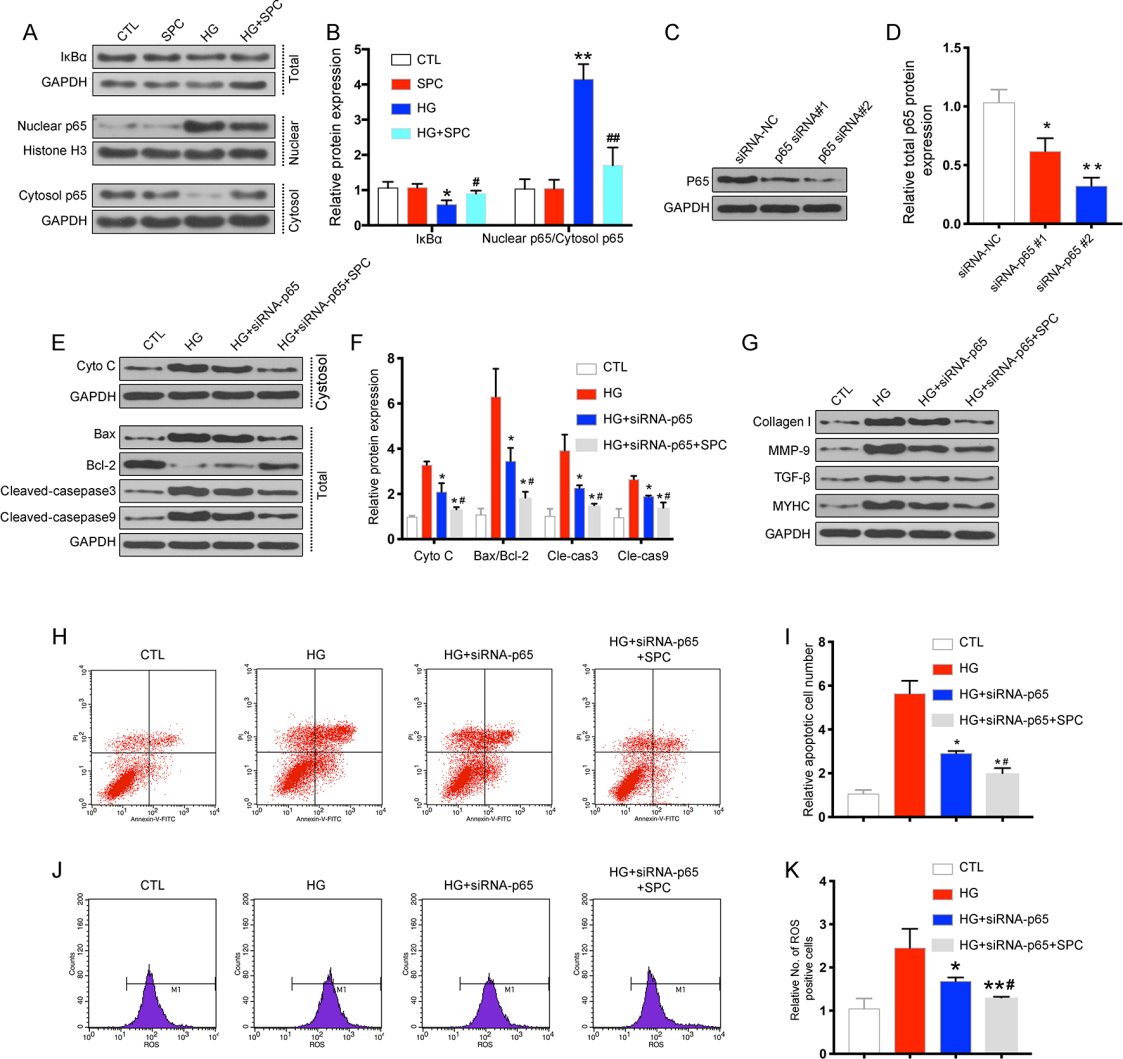
SPC suppressed the activation of the NF-κB signaling in HG-stimulated inflammatory responses in H9c2 cells. **(A)** The results of western blot analysis showed that incubation with HG for 12 h remarkblely induced IκBα degradation, and this alteration was significantly reversed by SPC treatment. HG stimulation markedly increased the nuclear translocation of NF-κB/p65, which was abolished by SPC treatment (1 mM). **(B)** Quantification of the western blot analysis. *P < 0.05 when compared with the results of control group; **P < 0.01 when compared with the results of control group; ^#^P < 0.05 when compared with the results of HG group; ^##^P < 0.01 when compared with the results of HG group. **(C** and **D)** The WB analysis showed successful knockdown of p65 using siRNAs. **(E**–**G)** WB analysis showed p65 knockdown using siRNA partially mimicked the anti-apoptotic and anti-inflammatory effects of SPC treatment. **(H** and **I)** The results of flow cytometry apoptosis assay. **(J** and **K)** The results of flow cytometry ROS production assay. *P < 0.05 when compared with the results of the HG group; **P < 0.01 when compared with the results of the HG group; ^#^P < 0.05 when compared with the results of the HG + siRNA-P65 group; ^##^P < 0.01 when compared with the results of the HG+siRNA-P65 group. CTL, control group; SPC, Sophocarpine; HG, high glucose; ROS, reactive oxygen species.

To further confirm the protective effects of SPC on HG-induced H9c2 cells were related with anti-inflammatory, the transcription factor NF-κB signaling pathway is investigated. First, we determined the effect of SPC on IκBα degradation in H9c2 cells incubated with HG. The results showed that incubation with HG (33 mM) for 12 h remarkably induced IκBα degradation, and this alteration was significantly reversed by SPC treatment (1 mM). Then, we analyzed the expression of p65 protein in both the nucleus and cytoplasm. HG stimulation markedly increased the nuclear translocation of NF-κB/p65, which was abolished by SPC treatment (**Figures 3A, B**).

To explore the possible mechanism underlying the regulation of SPC on Bcl-2 and Bax expression, small interfering RNAs (siRNAs) targeting p65 were used. As is shown in **Figures 3C, D**, the WB analysis showed successful knockdown of p65 using siRNAs. Based on the results of WB analysis, p65 siRNA#2 was chosen for subsequent experiments. As is shown in **Figures 3E–K**, p65 knockdown using siRNA partially mimicked the anti-inflammatory (**Figure 3G**) and anti-apoptotic effects (**Figures 3E, F, H, I**) and anti-oxidative stress (**Figure 3J**) of SPC, as SPC treatment (1 mM) together with p65 knockdown further augmented these effects. Thus, SPC treatment regulated the expression of Bcl-2 and Bax and protected against HG-induced cardiomyocyte apoptosis partially through the inhibition of NF-κB p65.

### SPC Treatment Attenuated Diabetes-Induced Cardiac Dysfunction

**Figure 4:**
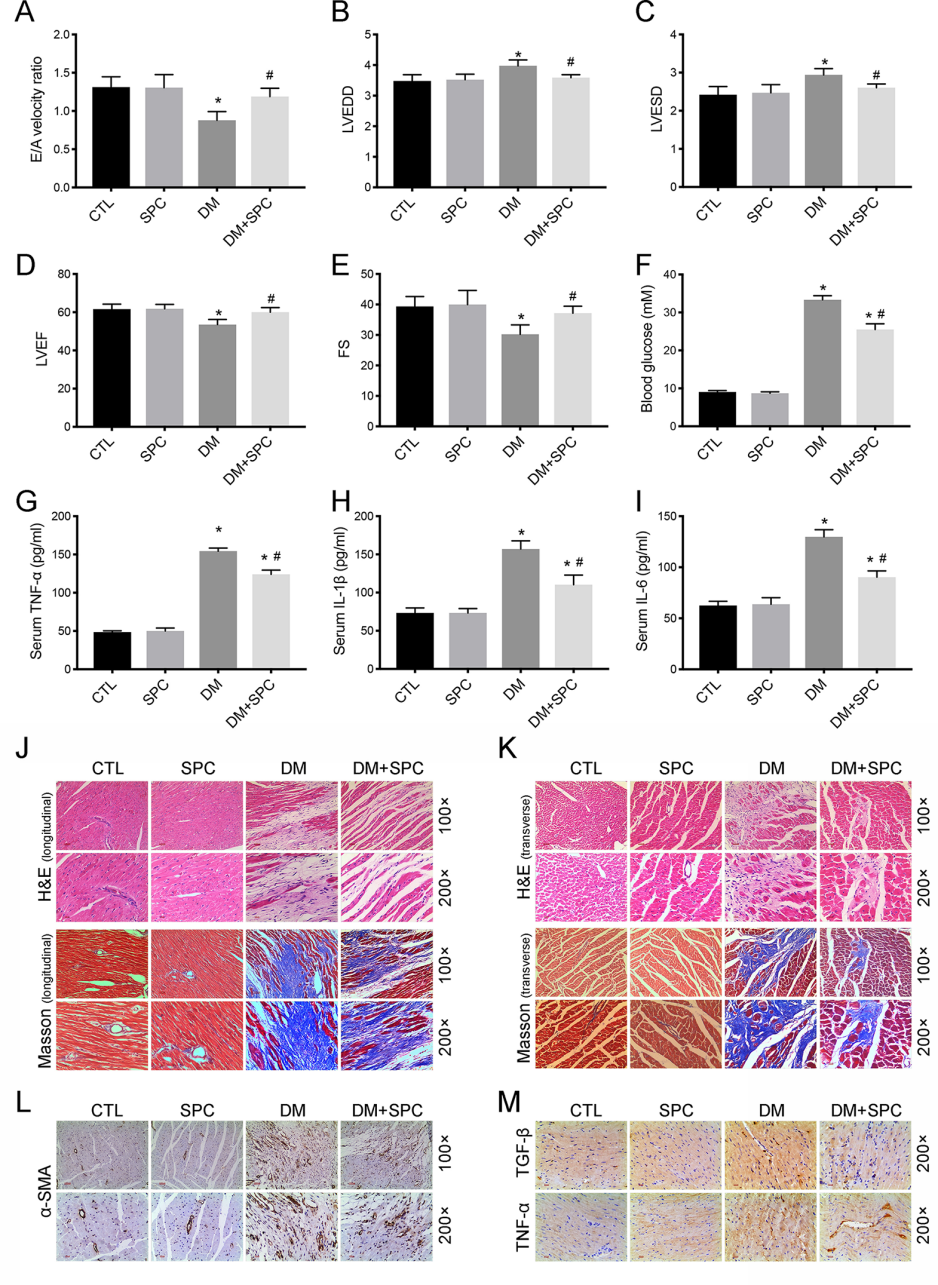
SPC treatment attenuated diabetes-induced cardiac dysfunction and cardiac remodeling. **(A**–**E)** The results of echocardiography demonstrated that mice in the DM group exhibited significant cardiac dysfunction compared to mice in the control group, with significantly decreased E/A velocity ratio, LVEF, FS, and increased value of LVEDD and LVEDD. After SPC treatment, all these parameters improved notably when compared to DM group. **(F)** The increased blood glucose level induced by DM was effectively inhibited after SPC treatment. **(G**–**I)** Serum inflammatory factor (TNF-α, IL-1β, IL-6) levels were also effectively inhibited after SPC treatment. **(J** and **K)** H&E staining and Masson trichrome staining (longitudinal view on the left and transverse view on the right) showed obvious structural abnormalities and collagen accumulation in the myocardial tissues from DM group, while SPC treatment remarkably reduced the collagen deposition and fibrosis. **(L)** The results of α-SMA IHC staining showed a significant increase of α-SMA protein expression in the DM group, and the protein expression level of α-SMA was decreased after SPC treatment. **(M)** The expression of TGF-β and TNF-α was also decreased after the treatment of SPC. E/A velocity ratio: the ratio of early to late mitral valve flow velocity E/A velocity ratio; LVEF, left ventricular ejection fraction; FS, percentage of fractional shortening; LVESD, left ventricular end-systolic diameter; LVEDD, left ventricular end-diastolic diameter; IHC staining, immunohistochemical staining; CTL, control group; SPC, Sophocarpine; DM, diabetes mellitus group. *P < 0.05 when compared with the results of control group; ^#^P < 0.05 when compared with the results of DM group.

Using a type 1 diabetic mouse model, we assessed the *in vivo* effect of SPC on DCM. As is shown in **Figures 4A–E**, the results of echocardiography demonstrated that mice in the DM group exhibited significant cardiac dysfunction compared to mice in the control group, with significantly decreased E/A velocity ratio, left ventricular ejection fraction (LVEF), fractional shortening (FS), and increased value of left ventricular end-diastolic diameter (LVEDD) and left ventricular end-systolic diameter (LVESD). After SPC treatment, all these parameters improved when compared to DM group. Moreover, the increased blood glucose level (**Figure 4F**) and serum inflammatory factor [tumor necrosis factor-α (TNF-α), interleukin-1β (IL-1β), IL-6] levels (**Figures 4G–I**) induced by DM were also effectively inhibited after SPC treatment. Taken together, these evidences suggested that SPC administration attenuated the diabetes-induced cardiac dysfunction.

### SPC Relieved Diabetes-Induced Cardiac Remodeling

**Figure 5:**
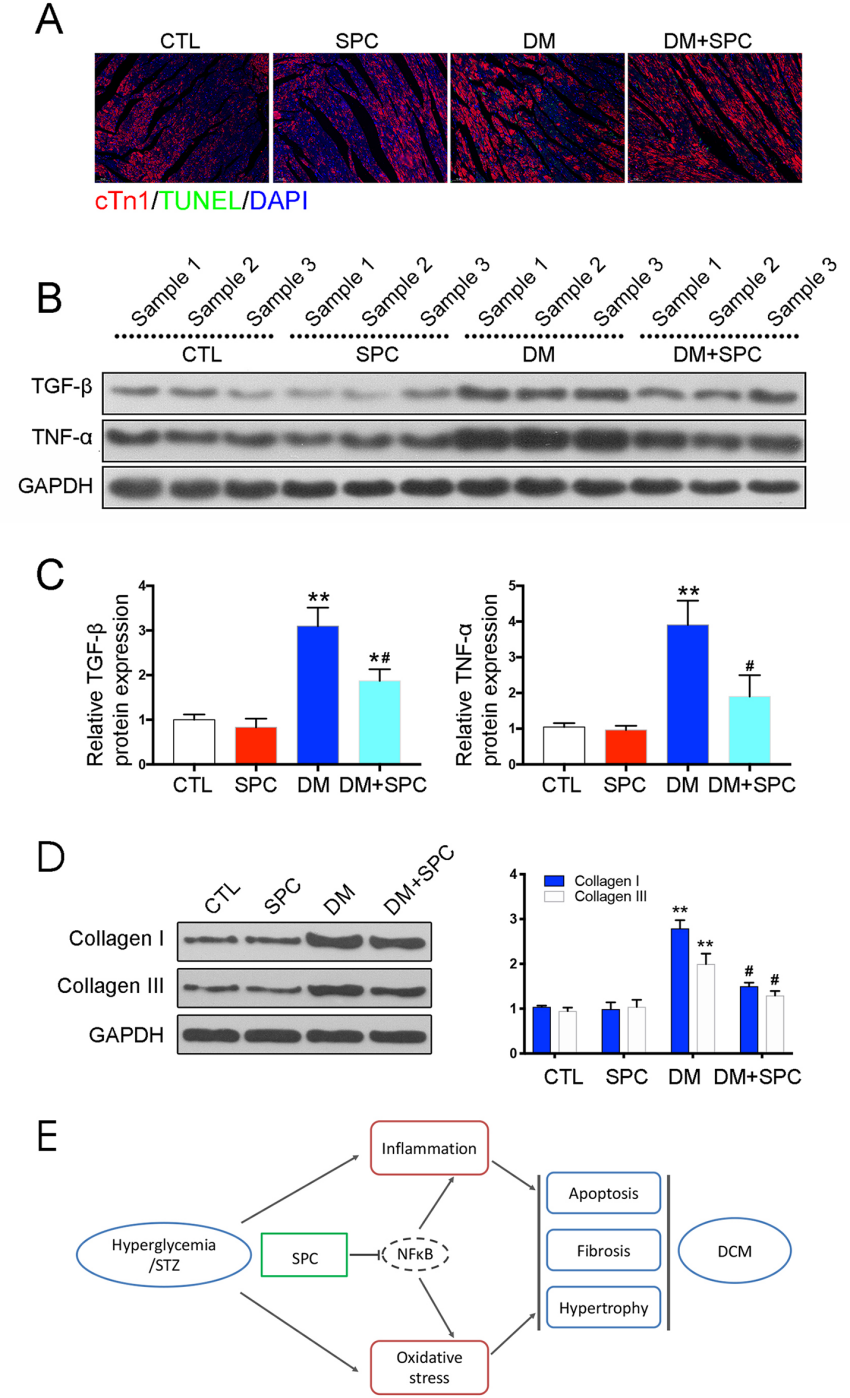
SPC mitigated diabetes-induced cardiac inflammation and myocardium apoptosis. **(A)** TUNEL staining showed significantly increased cell apoptosis (TUNEL-positive cells) in diabetic hearts, which was remarkably mitigated by SPC treatment. Figures are magnified as 100×. **(B)** The results of western blot analysis revealed a marked elevation in the protein expression of TNF-α in diabetic myocardial tissues compared with the control group. After SPC treatment, the protein expression of TNF-α was significantly reduced. **(C)** Quantification of the western blot analysis. **(D)** Heart tissue samples from mice in diabetes mellitus group have significantly higher Collagen I and Collagen III protein expression than samples from the control group, which was remarkably inhibited by SPC treatment. **(E)** A diagram recapitulating the main findings of this study. CTL, control group; SPC, Sophocarpine; DM, diabetes mellitus group. *P < 0.05 when compared with the results of control group; **P < 0.01 when compared with the results of control group; ^#^P < 0.05 when compared with the results of DM group.

We then applied histological analysis to find out the *in vivo* role of SPC on fibrosis and histopathology of diabetic hearts. As shown in **Figures 4J–K**, hematoxylin and eosin (H&E) staining and Masson trichrome staining showed obvious structural abnormalities and collagen accumulation in the myocardial tissues from DM group, while SPC treatment remarkably reduced the collagen deposition and fibrosis. Consistent with the evidence from H&E staining and Masson trichrome staining, the results of α-SMA IHC staining showed a significant increase of α-SMA protein expression in the DM group, which again indicated obvious fibrosis of myocardial tissues. As expected, the protein expression level of α-SMA was decreased after SPC treatment (**Figure 4L**). In addition, the expression of pro-fibrotic biomarker TGF-β was also decreased after the treatment of SPC (**Figure 4M** and **Figures 5B, C**). As is shown in **Figure 5D**, heart tissue samples from mice in diabetes mellitus group have significantly higher Collagen I and Collagen III protein expression than samples from the control group, which was remarkably inhibited by SPC treatment. Taken together, these results indicated that SPC treatment reduces collagen deposition and fibrosis, eventually contributing to relieve diabetes-induced cardiac remodeling.

### SPC Mitigated Diabetes-Induced Cardiac Inflammation and Myocardium Apoptosis

We next examined the role of SPC in alleviating inflammation and myocardium apoptosis in diabetic hearts. Consistent with the histomorphometric observation, the results of IHC staining and western blot analysis revealed a marked elevation in the protein expression of pro-inflammatory biomarker TNF-α in diabetic myocardial tissues compared with the control group. After SPC treatment, the protein expression of TNF-α was significantly reduced (**Figure 4I** and **Figures 5B, C**). We finally applied TUNEL staining to investigate the anti-apoptotic effect of SPC in diabetic myocardial tissues. We observed significantly increased cell apoptosis (TUNEL-positive cells) in diabetic heart samples, which was remarkably mitigated by SPC treatment (**Figure 5A**).

## Discussion

The results of this study demonstrated that SPC protected H9c2s cell from hyperglycemia-induced injury. SPC treatment also slowed the development and progression of DCM in streptozotocin (STZ)-induced diabetic mice. SPC was able to protect against DCM by improving mitochondrial function, suppressing inflammation, and inhibiting cardiac apoptosis. Moreover, SPC treatment inhibited the activation of NF-κB signaling in high-glucose-stimulated inflammatory responses, which suggests that SPC suppressed the inflammatory response and prevented cardiac dysfunction in diabetic mice by inhibiting the NF-κB signaling pathway (**Figure 5E**).

Several physiological factors are reportedly associated with the development of DCM, including cardiac inflammation, oxidative stress, and cardiac cell apoptosis. Among these, chronic and sustained inflammation is the major reason that hyperglycemia leads to changes in the structure and function of cardiac muscle (Lee et al., 2018; Yan et al., 2018; Ye et al., 2018; Ying et al., 2018; Althunibat et al., 2019). Hyperglycemia increases the expression of pro-inflammatory cytokines such as IL-6, IL-1β, TNF-α, and TGF-β. Indeed, anti-inflammatory therapeutic strategies have generally been beneficial in treating DCM (Ye et al., 2018; Zheng et al., 2018; Zhang et al., 2018a; Knapp et al., 2019). Zhang et al. reported that H3 relaxin protects against DCM by suppressing inflammation (Zhang et al., 2017). Feng et al. studied DCM in endothelial-specific microRNA (miR)-146a-overexpressing transgenic mice and found that miR-146a inhibits DCM by suppressing inflammatory changes (Feng et al., 2017). Similarly, deletion of the kinin receptor B1 gene reportedly slowed the development of DCM by suppressing cardiac inflammation (Westermann et al., 2009). Treatment with drugs that are based on natural products has some advantages over using artificial chemical compounds and genetic approaches. The clinical potential of drugs such as SPC may be enhanced if the cost is low, side effects are few, and availability is high.

The NF-κB signaling pathway plays a critical role in the pathology of DCM (Bombicz et al., 2019; Kang et al., 2019; Knapp et al., 2019). Hyperglycemia triggers IκBα degradation, allowing cytoplasmic NF-κB/p65 to be translocated into the nucleus. This promotes physiological processes associated with DCM including cell hypertrophy, apoptosis, and fibrosis. Recent studies have shown that inactivating the NF-κB pathway is an effective treatment for DCM (Li et al., 2011; Guo et al., 2018; Tang et al., 2018). In our study, hyperglycemia increased NF-κB activity and promoted cardiac apoptosis and fibrosis. These results are consistent with those from previous studies using H9c2 myocardial cells and STZ-induced diabetes mellitus models (Liu et al., 2017a; You et al., 2018). We also showed that SPC inhibited IκBα degradation and p65 translocation. In addition, our *in vivo* experiments demonstrated that myocardial apoptosis and fibrosis were significantly attenuated. Together, these data suggest that SPC protects against DCM at least partly by suppressing NF-κB-mediated inflammation.

Interestingly, other studies have shown that SPC has anti-inflammatory activity in various cell types and disease models. SPC decreased the expression of pro-inflammatory cytokines in both *in vitro* and *in vivo* experiments on chondrocytes, providing effective protection against osteoarthritis (Wu et al., 2019a). In mice hepatocytes, SPC inhibited lipopolysaccharide-induced septic liver injury by downregulating the NF-κB signaling pathway and suppressing inflammation (Jiang et al., 2018). SPC also inhibited the production of TNF-α and IL-6 in murine primary macrophages and prevented cachexia-related symptoms induced by colon 26 adenocarcinoma in mice (Zhang et al., 2008). In addition, SPC can stabilize prostheses by binding to IκB kinases, suppressing NF-κB/p65 activation and thus inhibiting osteoclast formation and implant loosening (Zhou et al., 2018). Our study is consistent with previous research and describes a novel method for treating DCM by using SPC. Our results may provide important information to support the clinical application of SPC.

This study had some limitations. First, the pathological processes associated with DCM are complex, and although we investigated the effects of SPC on inflammation, apoptosis, and myocardial fibrosis, we did not investigate other processes such as autophagy. Second, we did not demonstrate that SPC inhibits NF-κB signaling *in vivo*. Third, we did not apply extra osmotic cells group in which mannitol was added to keep the same osmolarity as that under conditions of HG. Thus, further studies will be needed to confirm the results of this study.

## Conclusions

Our study suggests that SPC has therapeutic potential and protects H9c2 cells from hyperglycemia-induced injury *in vitro*. Our mouse model experiments also show that SPC can be used to treat DCM *in vivo*. SPC may be effective against DCM because it can suppress inflammation and inhibit the NF-κB signaling pathway. These findings suggest that SPC may be effective in preventing DCM.

## Materials and Methods

### Reagents

SPC was purchased from Selleck Chemicals [Houston, USA; purity (%) = 99.80%]. It was dissolved in dimethyl sulfoxide (DMSO) and stored in the dark. The chemical structure of SPC is shown in **Figure 1A**.

### Cell Culture

The embryonic rat heart-derived cell line, H9c2, was obtained from ATCC (American Type Culture Collection, Manassas, VA, USA) and cultured in DMEM medium supplemented with 10% Fetal bovine serum (FBS), 100 mg/ml streptomycin, and 100 U/ml penicillin (Gibco, Waltham, MA, USA). Cells in the SPC treated group were pre-treated with SPC for 1 h and then exposed to high D-glucose concentration (33 mM, HG) or normal D-glucose (5.5 mM) DMEM medium, while cells in the control (CTL) group received same volume DMSO. The final concentration of DMSO in the medium of each group was less than 0.1% (v:v).

### Quantitative Real-Time PCR

Total RNA of H9c2 cells was extracted using TRIZOL (Invitrogen, Carlsbad, CA), following the procedure of standard protocol. Reverse transcription was performed using a Double-Strand cDNA Synthesis Kit (Takara, Dalian, China). qPCR was performed using a SYBR Green Master Mix kit (Takara). Primers were synthesized and obtained from Sangon Biotech (Shanghai, China). The details of primers used in this study were listed in **Table S1**.

### Cell Viability

H9c2 cells were seeded into 96-well plates at a density of 2×10^4^ cells/well for 24 h. After that, cells were treated with/without various doses of SPC (0.01–10 mM) for 48 or 96 h. After treatment, CCK-8 assay (Dojindo, Kumamoto, Japan) was carried out by adding 15 μl of CCK-8 buffer into each well. The plates were incubated 37°C for additional 4 h. The absorbance was detected at 450 nm on a microplate reader (Bio-Tek Instr., Winooski, VT, USA).

### Flow Cytometry Assay

Flow cytometry assay was performed to determine the apoptosis of H9c2 cells using an FITC Annexin V Apoptosis Detection Kit (BD Biosciences, USA). According to the manufacturer’s protocol, H9c2 cells were harvested and washed three times with phosphate buffer saline (PBS). After being incubated with FITC Annexin-V and propidium iodide (PI), cells were analyzed with a flow cytometry system (Becton Dickinson). In addition, ROS production was also measured by flow cytometry system (Becton Dickinson) using a DCFH-DA ROS assay kit (Sbjbio^®^ life science, Nanjing, China), according to the manufacturer’s instructions.

### Western Blot Assay

Cells or tissue samples were lysed with protein extraction reagent RIPA (Beyotime, Haimen, China), and protein amounts were assessed using a BCA-kit (Thermo fisher Scientific, MA, USA). In addition, a nuclear and cytoplasmic protein extraction kit (P00028; Beyotime) was applied to obtain nuclear and cytoplasmic protein samples. According to the manufacturer’s instructions, protein samples were then separated by SDS-PAGE gel and then transferred to polyvinylidene fluoride (PVDF) membranes (Millipore, MA, USA). After being blocked with 5% milk in TBST for 1.5 h at room temperature, the membranes were incubated overnight at 4°C with the following antibodies: COL-1 [1:1000, Cell Signaling Technology (CST), Danvers, MA, USA], MMP-9 (1:1000, Abcam, Shanghai, China), TGF-β (1:1000, CST), MyHC (1:1000, Abcam), B-cell lymphoma-2 (Bcl-2, 1:1000, Abcam), BCL2-associated X (Bax, 1:1000, CST), NF-κB/p65 (1:1000, CST), IkBα (1:1000, Abcam), cytochrome c (Cyto-C, 1:1000, Abcam), cleaved-caspase-3 (Cle-cas3), cleaved-caspase-9 (Cle-cas9), tumor necrosis factor-α (TNF-α, 1:1000, Abcam), glyceraldehyde-3-phosphate dehydrogenase (GAPDH, 1:1000, Abcam), and histone H3 (1:1000, CST) antibodies. Specific bands were detected by using an enhanced chemiluminescence kit (Bio-Rad, CA, USA) and were quantified by densitometry (Quantity One software, Bio-Rad).

### siRNA Construction and Infection

To explore the mechanism by which SPC inhibits NF-κB signaling pathway, two siRNAs against p65 and negative control (NC) were obtained from GenePharma (Shanghai, China). H9c2 cells were incubated with p65 siRNAs or the negative control siRNAs using standard methods. WB analysis was used to confirm the successful knockdown of p65.

### Isolation and Culture of NMCMs

NMCMs were prepared from newborn C57BL/6J mice (1–2 days old), as previously described (Brand et al., 2010; Xu et al., 2019). In brief, hearts of mice were cut into small pieces and digested with DMEM (Gibco) containing 0.03% trypsin and 0.03% collagenase II (Sigma). NMCMs were then isolated and cultured in DMEM with 20% FBS (Gibco) and 1% penicillin/streptomycin. After purification by 1.5 h differential preplating to allow cardiac fibroblast adherence, NMCMs were seeded onto dishes for 48 h and maintained in DMEM with 10% FBS and BrdU (0.1 mM, Sigma). After that, NMCMs were treated with HG and SPC, following the methods introduced above.

### Animal Experiments

The study protocols for all animal studies were approved by the Institutional Animal Care and Use Committee of the second affiliated hospital of Nanchang University and complied with the NIH guidelines for the care and use of laboratory animals. Male C57BL/6 mice (6–8 weeks old, weighing 18–22 g) used in this study were purchased from Animal Center of Nanchang University. In total, 40 mice were randomly divided into four groups (n = 10 each group): control group (CTL), SPC treatment group (SPC), diabetes mellitus group (DM), and DM plus SPC group (DM+SPC). Firstly, the DM mice were induced by daily intraperitoneal (i.p.) injection of 50 mg/kg STZ (dissolved in 100 mM citrate buffer, pH 4.5) for 5 consecutive days (Liu et al., 2017a; Tang et al., 2018), while mice in the CTL group and SPC group received same volume of sodium citrate buffer. One week later, mice with fasting blood glucose levels ≥250 mg/dl were defined as successful establishment of diabetes mellitus. Then, mice in the SPC and DM+SPC group received daily i.p. injection of SPC (20 mg/kg) (Li et al., 2011; Zhou et al., 2018), while mice in the CTL group and DM group received same volume of PBS. Sixteen weeks after the administration, blood glucose level was measured by tail vein blood using an automated analyzer (Beckman, CA, USA). After that, mice were euthanized under anesthesia, and heart tissues were collected and kept in liquid nitrogen or 4% paraformaldehyde for further analysis. Blood samples were collected from mice eye socket vEin. Serum was obtained by centrifugalization and kept at −80°C. Then, serum inflammatory factor (TNF-α, IL-1β, IL-6) levels were determined using enzyme-linked immunosorbent assay (ELISA) kits (BD Biosciences, CA, USA), according to the manufacturer’s protocol.

### Cardiac Function Assessment

Cardiac function was determined by M-mode echocardiography in anaesthetized mice 1 day before termination. An echocardiography system together with a 15-MHz linear transducer (VisualSonics Vevo 2100, Toronto, Canada) was applied. LVEF, percentage of FS [FS (%)], LVESD, LVEDD, and the ratio of early to late mitral valve flow velocity E/A velocity ratio (E/A velocity ratio) were measured using the machine by the same personnel.

### TUNEL Assay

The TUNEL apoptosis detection kit (R&D Systems, MN, USA) assay was also applied to detect cell apoptosis. H9c2 cells were washed three times with PBS and fixed with 4% paraformaldehyde, permeabilized with 0.1% TritonX-100, and then stained with TUNEL reagent. Following washing with PBS, cells were counterstained with DAPI (Sigma-Aldrich, USA) at room temperature for 5 min. For tissues, the sections of mice heart samples were treated for 60 min with TUNEL reagent (In Situ Cell Death Detection Kit; Roche Diagnostics) and DAPI for 5 min according to the manufacturer’s instructions. In addition, staining using a monoclonal antibody against Troponin I (cTnI, Santa Cruz) was also performed to identify the myocardium. Images were finally captured under a fluorescence microscope and quantified by Image J software. All morphometric measurements were conducted on five randomly selected fields for each sample.

### Histological Analysis

After being fixed in 4% paraformaldehyde for 48 h, mice heart samples were paraffin-embedded and sectioned at 5 µm thick for H&E, Masson trichrome staining, TUNEL staining, and immunohistochemical (IHC) staining as standard protocols. For IHC staining of α-SMA, TNF-α, and TGF-β, serial tissue sections were deparaffinized, rehydrated, and treated with 0.3% H_2_O_2_ for 30 min. After being blocked with 3% bovine serum albumin (BSA) in PBS for 30 min, the slides were then incubated with primary antibody at 4°C overnight (α-SMA, 1:200, CST; TNF-α, 1:100, Abcam; TGF-β, 1:250, Abcam). After that, a peroxidase-conjugated secondary antibody (Beyotime, China, 1:100) was applied for 1 h at room temperature. Finally, diaminobenzidine (DAB; Sigma-Aldrich, USA) was used to visualize the peroxidase binding sites and hematoxylin was used to visualize nuclears. Image-Pro Plus 6.0 (IPP 6.0) software was used to analyze the density of positive staining.

### Statistical Analysis

All data from at least three independent experiments were expressed as mean ± SDs. All statistical analyses were performed with SPSS 14.0 software (SPSS, Chicago, IL, USA). Statistical comparisons among different groups were evaluated using one-way ANOVA followed by multiple comparisons test with Bonferroni correction. *P* value <0.05 was considered statistically significant.

## Data Availability Statement

The datasets generated for this study are available on request to the corresponding author.

## Ethics Statement

The animal study was reviewed and approved by The Institutional Animal Care and Use Committee of the second affiliated hospital of Nanchang University.

## Author Contributions

Conception and design of the research: YS and RY. Acquisition of data: HL, LH, WW and XX. Analysis and interpretation of data: FZ and LWa. Statistical analysis: LWu. Obtaining funding: YS, FZ, and RY. Drafting the manuscript: FZ and LWa. Revision of manuscript for important intellectual content: YS and RY.

## Funding

This study was funded by National Natural Science Foundation of China (No. 81860151, 81560145, 81400815, and 81660063), National Key R&D Program of China (No. 2016YFC0900400), and Natural Science Foundation of Jiangxi province (20171BAB205041).

## Conflict of Interest

The authors declare that the research was conducted in the absence of any commercial or financial relationships that could be construed as a potential conflict of interest.
