# Supplementary material for: Sophocarpine Suppresses NF-κB-Mediated Inflammation Both In Vitro and In Vivo and Inhibits Diabetic Cardiomyopathy
Source: Front Pharmacol. 2019 Oct 31;10:1219. doi: 10.3389/fphar.2019.01219 (PMC6836764; doi:10.3389/fphar.2019.01219)
Supplement: Supplementary file 1 [file Image_1.pdf]

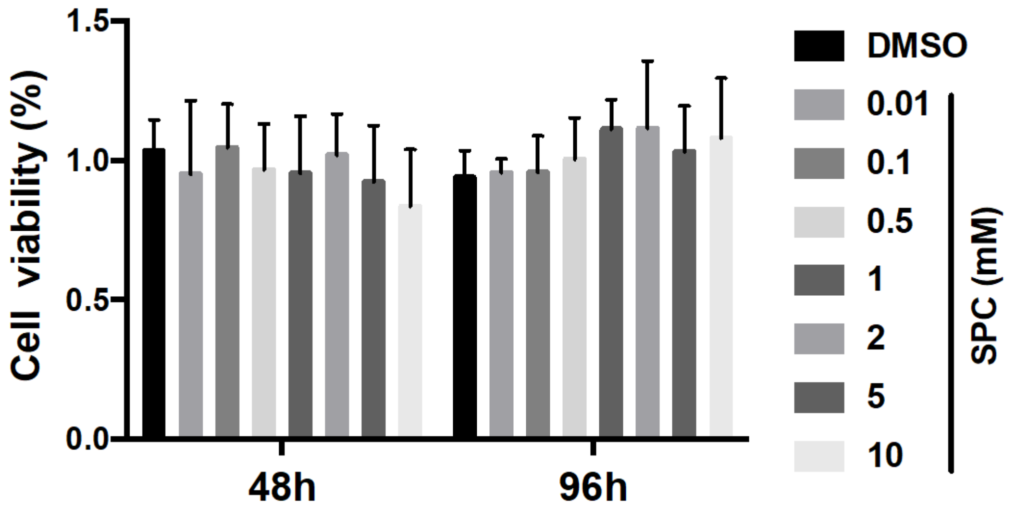

Supplement Figure 1. The effects of SPC on H9c2 viability. H9c2 cells treated with SPC at indicated dose, and subjected to CCK-8 assay after 48 and 96h treatment as described in Methods.
