# Supplementary material for: Sophocarpine Suppresses NF-κB-Mediated Inflammation Both In Vitro and In Vivo and Inhibits Diabetic Cardiomyopathy
Source: Front Pharmacol. 2019 Oct 31;10:1219. doi: 10.3389/fphar.2019.01219 (PMC6836764; doi:10.3389/fphar.2019.01219)
Supplement: Supplementary file 2 [file Image_2.pdf]

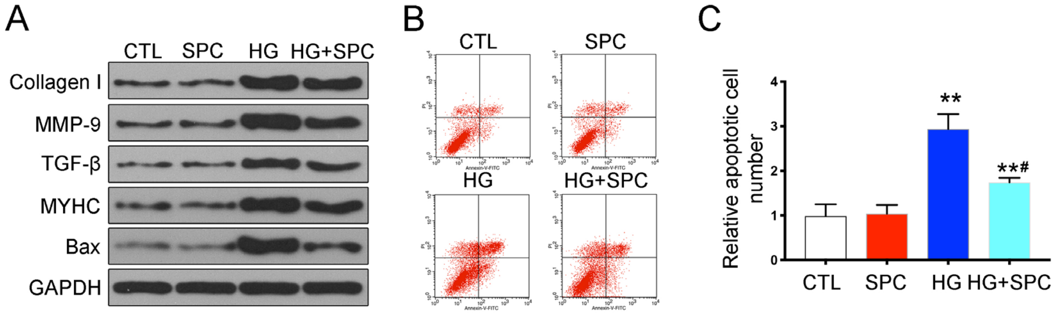

Supplement Figure 2. SPC attenuated HG-stimulated inflammatory responses and apoptosis in NMCMs. (A) Western blot analysis showed that HG stimulation for 12 hrs remarkebly increased the expression of COL-1, MMP-9, TGF-β, MyHC and Bax, which was then significantly inhibited by SPC (1μM). **(B-C)** Flow cytometry assay showed that the increased apoptosis of NMCMs by hyperglycaemia was effectively attenuated by SPC (1μM). CTL: control group; SPC: Sophocarpine; HG: hyperglycaemia. *P<0.05 when compared with the results of control group; **P<0.01 when compared with the results of control group; $^{\#}P$<0.05 when compared with the results of HG group; $^{\#\#}P$<0.01 when compared with the results of HG group.
