# Supplementary material for: Sophocarpine Suppresses NF-κB-Mediated Inflammation Both In Vitro and In Vivo and Inhibits Diabetic Cardiomyopathy
Source: Front Pharmacol. 2019 Oct 31;10:1219. doi: 10.3389/fphar.2019.01219 (PMC6836764; doi:10.3389/fphar.2019.01219)
Supplement: Supplementary file 3 [file Table_1.doc]

**Table S1:** Primers used for real-time qPCR assay.

| Gene | Forward (5’-3’) | Reverse (3’-5’) |
| --- | --- | --- |
| COL-1 | GACATCCCTGAAGTCAGCTGC | TCCCTTGGGTCCCTCGAC |
| TGF-β | GCAACAACGCAATCTATGAC | CCTGTATTCCGTCTCCTT |
| MyHC | CGAGTCCCAGGTCAACAAG | AGGCTCTTTCTGCTGGACA |
| MMP9 | AGGTGCCTCGGATGGTTATCG | TGCTTGCCCAGGAAGACGAA |
| 18S | AGTCGCCGTGCCTACCAT | CGGGTCGGGAGTGGGTAAT |
